# Depletion of TrkB Receptors From Adult Serotonergic Neurons Increases Brain Serotonin Levels, Enhances Energy Metabolism and Impairs Learning and Memory

**DOI:** 10.3389/fnmol.2021.616178

**Published:** 2021-04-15

**Authors:** Madhusmita P. Sahu, Yago Pazos-Boubeta, Anna Steinzeig, Katja Kaurinkoski, Michela Palmisano, Olgierd Borowecki, Timo Petteri Piepponen, Eero Castrén

**Affiliations:** ^1^Neuroscience Center, Helsinki Institute of Life Science HiLIFE, University of Helsinki, Helsinki, Finland; ^2^Faculty of Philosopy and Social Sciences, Nicolaus Copernicus University in Toruń, Toruń, Poland; ^3^Division of Pharmacology and Pharmacotherapy, University of Helsinki, Helsinki, Finland

**Keywords:** serotonin, TrkB, BDNF, neuronal plasticity, neurotrophic factor

## Abstract

Neurotrophin brain-derived neurotrophic factor (BDNF) and neurotransmitter serotonin (5-HT) regulate each other and have been implicated in several neuronal mechanisms, including neuroplasticity. We have investigated the effects of BDNF on serotonergic neurons by deleting BDNF receptor TrkB from serotonergic neurons in the adult brain. The transgenic mice show increased 5-HT and Tph2 levels with abnormal behavioral phenotype. In spite of increased food intake, the transgenic mice are significantly leaner than their wildtype littermates, which may be due to increased metabolic activity. Consistent with increased 5-HT, the proliferation of hippocampal progenitors is significantly increased, however, long-term survival of newborn cells is unchanged. Our data indicates that BDNF-TrkB signaling regulates the functional phenotype of 5-HT neurons with long-term behavioral consequences.

## Introduction

Neurotrophin Brain-derived neurotrophic factor (BDNF) and neurotransmitter serotonin (5-HT) regulate neuronal survival, neurogenesis, and neuronal plasticity and they also co-regulate each other (Mattson et al., 2004; Martinowich and Lu, 2008). Changes or aberrations in these two systems, together or independently, are associated with neuropsychiatric disorders, which are a major health problem worldwide (Homberg et al., 2014).

Brain-derived neurotrophic factor is associated with the regulation of activity-dependent neuronal connectivity and plasticity. BDNF together with its high-affinity cognate receptor, neurotrophic tyrosine kinase receptor 2 (Ntrk2/TrkB) plays a significant role in neuronal survival, synaptic plasticity, and in mediating 5-HT metabolism (Mamounas et al., 2000). BDNF and TrkB induce serotonergic phenotype and increase the number of 5-HT expressing neurons (Galter and Unsicker, 2000a). BDNF and TrkB have been concomitantly related together with 5-HT in a myriad of neurochemical and behavioral responses (Martinowich and Lu, 2008).

5-HT is a major modulatory neurotransmitter produced by neurons that are located in the brainstem raphe nuclei and project massively throughout the brain serving different physiological and behavioral functions (Dahlström and Fuxe, 1964; Steinbusch, 1981; Gaspar et al., 2003; Okaty et al., 2015). 5-HT is released both synaptically and through volume transmission (Halasy et al., 1992; Fuxe et al., 2007), and its effects are mediated by an ensemble of different receptors located on the target neurons as well as on 5-HT neurons themselves (Jacobs and Azmitia, 1992). Although a positive identification of BDNF and TrkB expression in serotonergic neurons is missing, TrkB are expressed in the region of raphe nuclei (Madhav et al., 2001; Lunden and Kirby, 2013; Adachi et al., 2017). However, the physiological role of the activation of TrkB in 5-HT neurons remains unclear.

Previous studies have demonstrated that BDNF controls the survival and maintenance of developing 5-HT neurons through an auto/paracrine loop mediated by its autoreceptors, mainly 5HT1a, followed by sequential activation of BDNF and TrkB (Galter and Unsicker, 2000b). The 5-HT neurons from the mid-brain (MB) project throughout the brain and exert trophic actions on the target cells by controlling their proliferation and differentiation (Lauder et al., 1982; Commons, 2016).

We recently found that TrkB plays a critical role in the maintenance of 5-HT and dopamine (DA) neurons in zebrafish brain (Sahu et al., 2019). Constitutive knockouts of both TrkB and BDNF are postnatally lethal in mammals (Erickson et al., 1996), so conditional and inducible transgenic mouse (TG) models have been utilized to delete TrkB in a regionally and temporally selective fashion. A recent study reported that deletion of TrkB from neurons in the midbrain raphe region resulted in a loss of antidepressant efficacy and heightened aggression (Adachi et al., 2017). In this study we have specifically deleted TrkB in Tph2 expressing 5-HT neurons in the adulthood, after the serotonergic system has fully matured, and demonstrate a key role for TrkB in serotonergic neurons in the regulation of 5-HT function. The effects of loss of TrkB in 5-HT neurons on behavior were also assessed by a battery of tests measuring anxiety, aggression, learning, and memory.

## Materials and Methods

### Animals

The Tph2creERT2 animals were obtained from Prof. Pierre Chambon laboratory (Feil et al., 1996; Yadav et al., 2011). The animals were rederived and backcrossed to the C57BL/6J strain in our animal facility for several generations. The TrkBflox mice were obtained from the Jackson lab and have been maintained in our system with C57BL/6J background.

The Tph2creERT2 mice were crossed with homozygous TrkBflox mice to generate the Tph2creERT2:TrkBflox mice in the C57BL/6J strain background. The cre is hemizygous and TrkBflox is homozygous, therefore every internal mating produced Tph2creERT2:TrkBflox and TrkBflox mice. The animals that were cre positive have been grouped as transgenic and the flox alone as control throughout this manuscript. The cohorts were made by pooling several littermates born at the same time.

For cre activation, tamoxifen was dissolved in corn oil with continuous shaking at 37°C overnight. The tamoxifen stock was made into a final concentration of 20 mg/ml. For all the experiments we have used the same injection time. Six weeks old mice were administered tamoxifen at 0.1 mg/kg intra peritoneally (ip) dose daily for 5 days to activate the cre-mediated recombination. The control and transgenic mice that had received tamoxifen are called Ctrl and TG, respectively. The transgenic animals not injected with tamoxifen are represented as TG w/o TAM.

For reporter assay, tDtomato mice (JAX 007914, Jackson Lab) were crossed with Tph2creERT2 animals. The Tph2creERT2:tDtomato animals were divided into two groups, one receiving tamoxifen and others received only corn oil. All the animals were maintained according to the guidelines from the ethical committee of Southern Finland (License number- ESAVI/10300/04.10.07/2016).

### Behavioral Tests

Adult animals aged between 6 and 9 months were used for all the behavioral tests. Mice were single housed during the test period with unlimited access to food and water in the individually ventilated cage system. The single housing was done 1 week prior to the start of the behavior tests for a maximum of 1 month. Mice were kept at light/dark rhythm of 12 h each. The number of animals tested was 60 in total, with 30 experimental and 30 control group unless otherwise mentioned. The animals for most of the behavioral battery tests were males. The animals were always transferred to test rooms to adapt 30 min before starting the test. All behavioral tests were performed with the researcher blinded to the genotypes of the animals. We used three different cohorts of animals in this study. Therefore, in the first cohort we performed open field, light-dark, elevated plus maze and forced swim tests as well as Comprehensive lab animal monitoring system test, in the second cohort Barnes maze and resident intruder test, and in the third cohort the pattern separation test. The interval between the tests was usually 2 days for less stressful and 1 week for stressful experiments.

#### Open Field Test (OF)

The open field test was done with a brightly illuminated (550 lux) polypropylene chamber (30 × 30 × 30 cm). One mouse was put into each of the chamber for 30 min and their movement was monitored by Activity monitor (Med Associates Inc., United States). In this test, the total time of locomotor activity was recorded. The total ambulatory distance traveled was compared between the groups. The number of animals used were *N* = 30/group.

#### Light-Dark Test (LD)

The light–dark test was done in polypropylene chamber (30 × 30 × 30 cm), equipped with infrared light sensors detecting horizontal and vertical activity. A dark insert was used to divide the arena into two compartments. An opening on the wall of the dark insert ensured free movement of the animal between the two compartments. Illumination in the center of the light area was ∼550 lux. The mice were placed into the dark area at the start of the experiment. Their movements in and between the two areas were recorded and the data was collected with this activity monitoring setup for 30 min. Parameters for analysis were time spent in different compartments, vertical counts on both of the sides, latency to dark and total distance moved. The number of animals used were *N* = 30/group.

#### Elevated Plus Maze (EPM)

The elevated plus maze consisted of two open arms (30 × 5 cm) and two enclosed arms (30 × 5 cm) connected by a 5 × 5 cm central arena. The whole platform was 40 cm above the floor. The floor of the arms was light gray. The closed arms had transparent walls 15 cm high. The illumination during the test was ∼150 lux. The animal was placed in the center of the maze and tracked for a total of 5 min. The animals were tracked using Noldus EthoVision XT10 system (Noldus Information Technology, Netherlands). The parameters analyzed were latency to open arms, number of entries to the open and closed arms. The number of animals used were *N* = 30/group.

#### Comprehensive Lab Animal Monitoring System (CLAMS)

For metabolic monitoring, animals were subjected to comprehensive lab animal monitoring system (CLAMS) (Columbus Instruments, United States) for 3 days. This included 1 day of acclimatization and 2 days for data collection. The number of animals used were *N* = 12/group.

#### Resident-Intruder Test (RI)

The resident intruder test was performed in the animal’s home cage and the trial was video recorded. The experimental animals were single-housed, and the intruder animals were group housed. This experiment was performed in the experimental animals (resident) home cage. The duration of the test was 10 min and the parameters analyzed were number of attacks by the resident animal, time of social interaction measured as duration spent on sniffing, chasing, climbing and non-social exploration such as digging, rearing, grooming, and scanning the intruder as it moves away. The total number of animals used were *N* = 11/group.

#### Forced Swim Test (FST)

The mice were injected with fluoxetine 30 mg/kg intra peritoneally (ip) or saline ip 30 min prior to the test. A mouse was placed into a glass cylinder filled with tap water stabilized to room temperature. The water was filled to the height of 14 cm in the glass cylinder. The animals were tracked using the Noldus EthoVision XT10 system (Noldus Information Technology, Netherlands). The immobility time was measured during the 6-min testing period. The number of animals used were *N* = 12/group.

#### Pattern Separation (PS) Test

The pattern separation test was based on a published protocol (Diaz et al., 2013) that uses a contextual fear discrimination learning paradigm. Both males and females were used in this paradigm. Males were tested first followed by females to avoid any discrepancies. The animals were subjected to two contexts A and B, which were highly similar to each other and subjected for the same duration of time. Context A was the training chamber and it referred to a fearful episode where the mice received a single 2 s foot shock of 0.8 mA at 180 s after being placed in the chamber. Context B was highly similar, except for a mild vanilla smell in the bedding and two different patterned paper inserts in the chamber wall. The test was carried out for 10 consecutive days. On test days 1, 4, 7, 8, and 10, mice were first exposed to context A before context B, and on test days 2, 3,5, 6, and 9 they were first exposed to context B followed by context A. The percentage of freezing in context A vs. B was evaluated on the last day. The number of animals used were *N* = 20/group.

#### Barnes Maze Test (BM)

The Barnes Maze test was conducted based on the published protocol for a period of 6 days (Harrison et al., 2006). The acquisition training was done for 3 days with three trials per day and the inter-trial interval was for 1 h. On the 4th and 6th day the probe trial was performed. Reversal training was started on the 4th day, after the first probe trial. The latency to find the escape box was measured during training and time spent in the vicinity of the target hole was measured during probe trials. The number of animals used were *N* = 12/group.

### High Pressure Liquid Chromatography (HPLC)

The brains were dissected from adult animals after terminal anesthesia with CO_2_. We used naïve animals, *N* = 4–5 per group. The different regions of the brain collected for analysis were the pre-frontal cortex (PFC), striatum, hippocampus (HC), hypothalamus, mid-brain (MB), and lower brain stem (LBS). The different brain structures were weighed, collected into tubes and frozen on dry ice. The samples were homogenized in 0.3 ml (pre-frontal cortex, striatum, hippocampus, hypothalamus) or 0.4 ml (mid-brain and lower brain stem) of homogenization solution consisting of six parts 0.2 M HClO_4_ and one part antioxidant solution (1.0 mM oxalic acid, 0.1 M acetic acid, 3.0 mM L-cysteine) (Kankaanpaa et al., 2001) with Rinco ultrasonic homogenizer (Rinco Ultrasonics AG, Romanshorn, Switzerland). The homogenates centrifuged at 20,800 *g* for 35 min at 4°C. The supernatant was passed through 0.5 ml Vivaspin filter concentrators (10,000MWCO PES; Sartorius, Stonehouse, United Kingdom) and centrifuged at 8,600 *g* at 4°C for 35 min. The medium collected from the cell cultures were filtered and processed likewise.

Filtrates containing monoamines were analyzed using high-pressure liquid chromatography with electrochemical detection. Analyses of dopamine (DA), serotonin (5-HT) and its main metabolite 5-hydroxyindoleacetic acid (5-HIAA) were performed. The analytes were separated on a Phenomenex Kinetex 2.6 μm, 4.6 × 100 mm C-18 column (Phenomenex, Torrance, CA, United States). The column was maintained at 45°C with a column heater (Croco-Cil, Bordeaux, France). The mobile phase consisted of 0.1 M NaH_2_PO_4_ buffer, 120 mg/l of octane sulfonic acid, methanol (5%), and 450 mg/l EDTA, the pH of mobile phase was set to three using H_3_PO_4_. The pump (ESA Model 582 Solvent Delivery Module; ESA, Chelmsford, MA, United States) was equipped with two pulse dampers (SSI LP-21, Scientific Systems, State College, PA, United States) and provided a flow rate of 1 ml/min. One hundred microliters of the filtrate was injected into chromatographic system with a Shimadzu SIL-20AC autoinjector (Shimadzu, Kyoto, Japan). Monoamines and their metabolites were detected using ESA CoulArray Electrode Array Detector with 12 channels (ESA, Chelmsford, MA, United States). The chromatograms were processed, and the concentrations of monoamines were calculated using CoulArray for Windows software (ESA, Chelmsford, MA, United States). The concentrations of analytes are expressed as ng/g of wet tissue.

### Western Blotting

The brains from both males and females were dissected out from adult animals (*N* = 4/group). The different brain structures used for analysis were the hippocampus, hypothalamus, and mid-brain. The brain samples were homogenized using the standard RIPA lysis buffer, containing a cocktail of protease inhibitors and orthovanadate, followed by centrifugation at 13,000 rpm for 15 min at + 4°C. The Rn33b cells were lysed similarly and sonicated before centrifugation. The samples were then separated using gradient gels 4–15% (NuPAGE Protein gels, Invitrogen) and blotted on to a PVDF membrane. The primary antibodies were RD-TrkB (Cat# AF397, RD systems Inc., MN, United States) and GAPDH (Cat# ab75479, Abcam). The primary antibodies were used at 1:1,000 dilution. The respective HRP conjugated secondary antibodies was blocked for 1 h at room temperature. The chemiluminescent assay was performed using ECL (Pierce). All images obtained were analyzed using ImageJ software.

### Proliferation and Survival of Hippocampal Cells

Proliferation and survival of newborn cells in the dentate gyrus (DG) was quantified using a dot-blot method that was performed as previously described, with minor modifications (Wu and Castrén, 2009; Casarotto et al., 2021). The animals used for the assessment of cell proliferation and cell survival assay were 9–11 months old (*N* = 4/group). Mice were injected with BrdU 75 mg/kg body weight four times at 2 h interval. For the proliferation study, the injection was done 24 h prior to sacrifice and for the survival study, the injection was done 4 weeks before the sacrifice. The animals were sacrificed 1 day or 4 weeks after BrdU administration, with terminal anesthesia using CO_2_, and brains were quickly removed. Hippocampi were dissected on ice, instantly frozen on dry ice and stored at −80°C until further use. DNA was isolated from one the of the hippocampi using DNeasy^®^ Blood and Tissue Kit (QIAGEN, Germany) according to the manufacturer’s instruction. The DNA purity was assessed by the spectrophotometer NanoDrop 2000C (Thermo Fisher Scientific, United States). DNA was incubated with 1 volume of 4 N NaOH solution for 30 min at room temperature to render it as single stranded and immediately kept on ice to prevent reannealing. The DNA solution was neutralized by an equal volume of 1 M Tris–HCl (pH = 6.8). The single-stranded neutralized DNA (1 mg) was pipetted in triplicates onto a nylon transfer membrane (Schleicher and Schuell, Keene, NH, United States) with a dot-blot apparatus (Minifold, Schleicher and Schuell) under vacuum and the DNA was fixed by ultraviolet cross-linker (1,200 μJ × 100, Stratagene, La Jolla, CA, United States). The membranes were incubated with mouse anti BrdU monoclonal antibody (1:1,000, B2531, Sigma) as the primary antibody and anti-mouse horseradish peroxidase (HRP) conjugated (Bio-Rad, United States) as the secondary antibody. The Pierce ECL Plus kit (Thermo Fisher scientific, United States) was used as a chemiluminescent method to develop the membrane. The membranes were scanned by imaging using a Fuji LAS-3000 Camera (Tamro Medlabs, Finland) and the densitometry analysis was performed by ImageJ Software.

### Immunohistochemistry and *in situ* Hybridization

A mixed cohort of animals were used for immunohistochemistry. They were 9–11 months old at the time of processing. Except for the reporter mice we used for checking cre specificity were 3 months old. They were terminally anesthetized with pentobarbital (Mebunat vet 60 mg/ml) and Lidocaine followed by transcardial perfusion with 4% PFA. The brains were stored in 4% PFA overnight at + 4°C and later transferred to 30% sucrose in PBS. These brains were cryo-embedded in an embedding matrix and stored at −80°C until further use. The brains were sectioned into 40 μm thick slices and stored in cryoprotectant solution at −20°C. For BrdU labeling, DNA was denatured by incubating the sections for 30 min in 2 M HCl at + 37°C and then 15 min in 0.1 M boric acid at room temperature. All the samples were processed for immunostaining as described earlier (Karpova et al., 2011).

The primary antibodies used in this study were Calretinin (Rabbit 7697, Catalog number CR 7697, Swant Switzerland), Doublecortin (Catalog number 4604, Cell signaling technology), BrdU (Catalog number ab82421, Abcam, United Kingdom), NeuN (Catalog number MAB377X, Millipore), GFAP (Catalog number 12389, Cell Signaling Technology), and Tph2 (Catalog number PA1-778, Thermo Fisher Scientific). The respective secondary antibodies were Alexa conjugated antibodies (Invitrogen). All the sections were stained with Hoechst 33342 (Thermo Fisher Scientific) before mounting. Stacked images were obtained using a 25× objective on a Zeiss confocal microscope LSM 780 with 1 μm interval between the sections. To avoid cross talk between channels in double labeled samples, we used sequential scanning. The cell counting and quantitation was done with the experimenter blinded to the treatment groups. The cell counting was performed as mentioned in Karpova et al. (2011) using ImageJ software (ImageJ 1.51s version) (Karpova et al., 2011). The images were collected in stacks and cell counting was done in each stack ensuring no overlap in between the stacks, although a stereological counting was not performed. The number of cells were averaged for every stacked image. For sample processing, we used at least five sections per hemisphere per animal and *N* = 4/group. The cell counting results are expressed as percentage to the control group.

For *in situ* hybridization, brains were collected on super-frost slides (*N* = 3/group) and processed with the riboprobe synthesized for *Bdnf* (Hofer et al., 1990). The sections were probed with both sense and antisense probe labeled with digoxigenin. After washing the probes, they were labeled with alkaline phosphatase conjugated anti-digoxigenin fab fragments (1:5,000, Roche Diagnostics, Germany) overnight. The probes were detected by a chromogenic substrate nitroblue tetrazolium/bromochloroindoyl phosphate (NBT/BCIP, Roche, Germany). The reaction was stopped, and brightfield images were obtained by a Nikon stereomicroscope.

### q-RT PCR

In this experiment we used *N* = 7 animals per group from a mixed cohort. The regions of interest including mPFC, hippocampus and midbrain were dissected and processed immediately for RNA isolation using the PureLink^®^ RNA Mini Kit (Thermo Fisher Scientific, United States). Reverse transcription of RNA was carried out using the SuperScript IV reverse transcriptase enzyme (Invitrogen/Thermo Fisher Scientific, United States). The CFX96 Touch Real-Time PCR detection system (Bio-Rad, United States) with SYBR Green fluorescent DNA probe (Thermo Fisher Scientific, United States) was used to perform the real time PCR. The data were calculated by the normalization of the expression using Ct values of a housekeeping gene (Hprt) as the reference control. The primers used were as follows:

*Hprt* F: GGGCTTACCTCACTGCTTTCC
*Hprt R*: CTAATCACGACGCTGGGACTG
*5ht2b* F: CCATTTCCCTGGACCGCTAT
*5ht2b* R: GGCGATGCCTATTGAAATTAACCA
*Tph2* F: AGAGTTGGAGACGGAGTCGT
*Tph2* R: AAGGGCAGTGGCTTATGACC
*Bdnf* F:CGATGCCAGTTGCTTTGTCTTC
*Bdnf* R:AGTTCGGCTTTGCTCAGTGG

### Statistics

All the experiments were analyzed using GraphPad Prism software version 9.0 (GraphPad Software Inc., CA, United States). Student’s *t* test (two-tailed) was used when two groups were compared. For more than two groups, the analyses performed were one-way or two-way analysis of variance (ANOVA) followed by Tukey’s *post hoc* test. All the error bars represent mean ± standard error of the mean (SEM) unless specified otherwise. The exact *p* values are mentioned in the text. The significance value was accepted at *p* ≤ 0.05.

## Results

### Deletion of TrkB in the Tph2 Neurons Increases 5-HT Production

**FIGURE 1:**
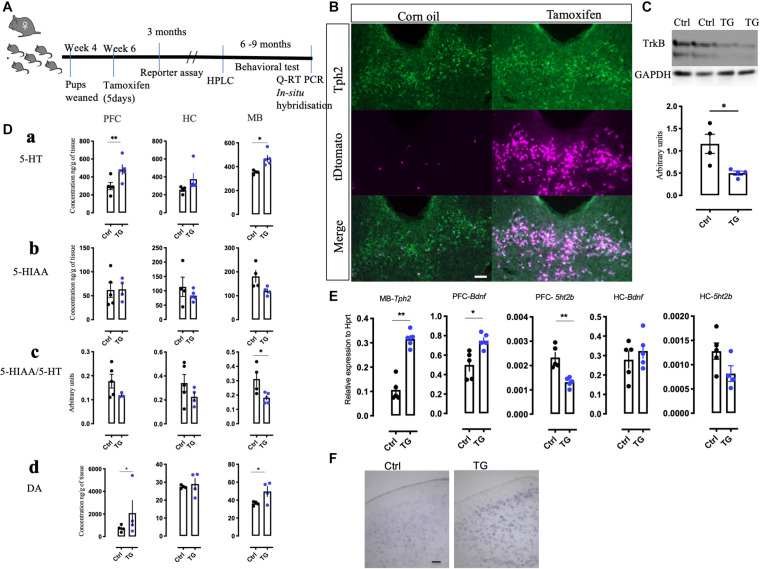
Generation of conditional knockout mice, reporter expression and alteration of 5-HT and its metabolite in the transgenic mice. **(A)** An experimental scheme representing the time of injection, reporter assay, HPLC measurements and the duration when different behavioral battery tests were performed followed by molecular analysis. **(B)** Confirmation of Cre-recombination in the tDtomato reporter mice using immunohistochemistry with Tph2 antibody in tamoxifen injected and corn oil injected animals (*N* = 4). **(C)** Western blotting with TrkB monoclonal antibody on the mid-brain samples from the control and TG mice (*N* = 4). **(D) (a)** The total levels of 5-HT was measured by HPLC in naïve animal’s different brain regions such as the PFC, HC, and MB. The levels in TG mice were significantly upregulated in the PFC and MB. **(b)** The levels of 5HIAA did not change in these brain regions. **(c)** The 5HIAA/5-HT ratio was also measured in these regions. The ratio was significantly down regulated in the MB of the TG mice. **(d)** The levels of DA were significantly upregulated in the PFC and MB of the TG mice (*N* = 4). **(E)** q-RT PCR results of the transcripts *Tph2* in the MB and *Bdnf* in the PFC was increased. The transcript for *5ht2b* was reduced in the PFC of the TG mice. No change in *Bdnf* and *5ht2b* transcripts in the HC was observed (*N* = 4). **(F)** *In situ* hybridization with *Bdnf* probe in the PFC of Ctrl and TG mice shows a robust expression (*N* = 3). Scale bar represents 100 μm, Student’s *t*-test, *p-value < 0.01, **p-value < 0.01. HPLC-High pressure liquid chromatography, 5-HT- Serotonin, 5HIAA- 5-Hydroxyindoleacetic acid, DA-Dopamine, PFC-pre-frontal cortex, HC-Hippocampus and MB-Mid-brain.

The timeline for all the experiments is summarized in Figure 1A. The specificity and effectiveness of creERT2-mediated recombination was verified by crossing the Tph2creERT2 mice with tDtomato reporter mice. One month after tamoxifen administration, tDtomato expression was observed to co-localize with the Tph2 antibody in the raphe nuclei of the mouse brain (Figure 1B and Supplementary Figure 1). In the control mice injected with corn oil, very few tDtomato expressing cells were visible and they were not colocalized with Tph2 antibody. This suggests that cre expression is activated with tamoxifen and is specific to the Tph2 specific serotonergic neurons.

For confirmation of TrkB deletion, brain tissues from the Tph2creERT2:TrkBflox (TG) and TrkBflox (Ctrl) mice were analyzed by western blotting. In the MB samples, where the 5-HT neurons are mostly located, the levels of TrkB were found to be reduced only in the cre- positive animals (*p* = 0.0239) (Figure 1C). A reduction, but not a total loss of TrkB signal was expected, since although 5-HT neurons are enriched in the MB regions, they nevertheless constitute a minority of all the TrkB positive cells in this region. No changes in TrkB levels were observed in the HC and hypothalamus (Supplementary Figure 2A).

Our previous study in zebrafish indicated that TrkB regulates 5-HT and DA neurons (Sahu et al., 2019). We therefore measured the levels of 5-HT, DA, and 5-HT metabolite 5-Hydroxyindoleacetic acid (5HIAA) in the MB containing the raphe nuclei, as well as in the projection regions of serotonergic neurons in the PFC and HC using HPLC. The levels of 5-HT were significantly increased in the MB and PFC and there was a trend toward increase in the HC (Figure 1Da). However, the levels of 5-HIAA were not significantly altered (Figure 1Db) and, consequently, the levels of 5-HIAA/5-HT ratio, a measure of 5-HT turnover was significantly reduced in the MB (Figure 1Dc). Furthermore, we found that the levels of DA were also increased in the MB as well as in the PFC in the TG animals (Figure 1Dd). The other brain regions without any significant changes are represented in Supplementary Figure 2B.

Consistent with increased 5-HT levels, q-RT-PCR experiments indicated that mRNA levels of the 5-HT synthetizing enzyme *Tph2* were significantly up-regulated (*p* = 0.0070) in the MB, which suggests that the capacity for 5-HT synthesis is increased in the transgenic animals (Figure 1E). We also investigated the mRNA levels for 5-HT receptors and found a significant decrease in the expression of *5ht2b* receptor in the PFC (*p* = 0.0022). No significant changes were found in the expression of other 5-HT receptors assayed, including *5ht1 (a,b,d), 5ht2 (a,c), 5ht3a, 5ht6*, and *5ht7* receptors (data not shown). Furthermore, the q-RT PCR (Figure 1E) indicated that *Bdnf* mRNA levels were upregulated in the PFC of the TG animals (*p* = 0.0355). A representative image of the *in situ* hybridization suggests increased reaction with the *Bdnf* probe in the TG animals (Figure 1F). Interestingly, we did not see any significant changes in the mRNA levels of *Bdnf* and *5ht2b* in the HC. Taken together, these results indicate that TrkB signaling significantly modulates the neurotransmitter phenotype of 5-HT neurons.

### Mice With Reduced TrkB in 5-HT Neurons Are Lean in Spite of Increased Food Intake

**FIGURE 2:**
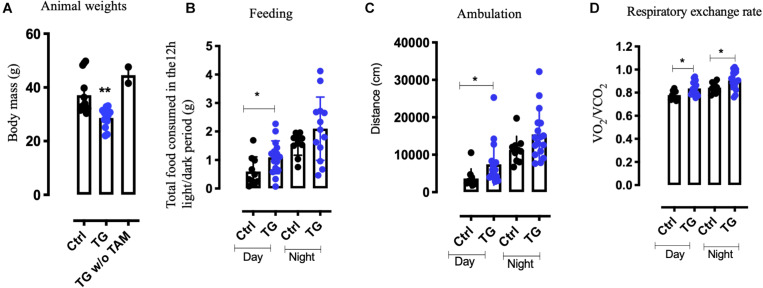
Increased metabolism and a lean phenotype is observed in the transgenic animals. **(A)** Weights of tamoxifen injected (TG) *N* = 10, tamoxifen uninjected (TG w/o TAm) *N* = 4 and control animals (Ctrl) *N* = 10, at 11 months of age. **(B)** D Metabolic readings using CLAMS suggest increase in food intake **(B)** and increased activity **(C)** in the TG mice. **(D)** The respiratory exchange rate was higher both during day and night in the TG mice (*N* = 12). One-way ANOVA, Student’s *t*-test, *p-value < 0.01, **p-value < 0.001, error bars represent Means ± SEM. CLAMS- comprehensive lab animal monitoring systems.

The TG littermates were found to be lean compared to the controls and transgenic mice not exposed to tamoxifen (*p* = 0.0067, Figure 2A). We therefore assessed the metabolic activity of the TG mice by subjecting the mice to the Comprehensive Laboratory Animal Monitoring System (CLAMS) for 3 days. Unexpectedly, we observed a significant increase in the feeding behavior (*p* = 0.0351) during daytime and a trend toward increase during nighttime. At the same time, locomotor activity was also increased (*p* = 0.0221) during daytime for the TG animal, again with a trend toward increase in nighttime activity (Figures 2B,C). Furthermore, the respiratory exchange rate (the ratio of the amount of CO_2_ expelled and O_2_ consumed) was elevated both during the day and night (Figure 2D) in the TG animals, indicating increased metabolic activity. These data suggest that, in spite of increased food intake, enhanced physical and metabolic activity led to a significant reduction in body weight of the TG mice.

### Behavioral Effects of TrkB Deletion in Adult Serotonergic Neurons

To assess the behavioral effect of TrkB loss in serotonergic neurons, we subjected both TG and Ctrl animals to a battery of behavioral tests, including tests for anxiety such as elevated plus maze (EPM), light dark test (LD) and open field (OF). For measuring aggression, we used the resident intruder (RI) test. The effect of Fluoxetine was studied using forced swim test (FST). The learning and memory were assessed using Pattern separation (PS) and Barnes maze (BM) test. These tests were performed in different cohorts of animals. The first cohort was exposed to OF, LD, EPM, CLAMS, and FST, in this order. There were 30 animals per group for OF, EPM, and LD. In the CLAMS and FST test we used *N* = 12/group. The second cohort was exposed to RI and BM and the *N* = 12/group. The third cohort was used for PS test and *N* = 20/group. The most stressful tests such as RI, FST, CLAMS, and PS test were performed in different cohorts of animals to reduce stress and variability arising from repeated exposures (Voikar et al., 2004).

**FIGURE 3:**
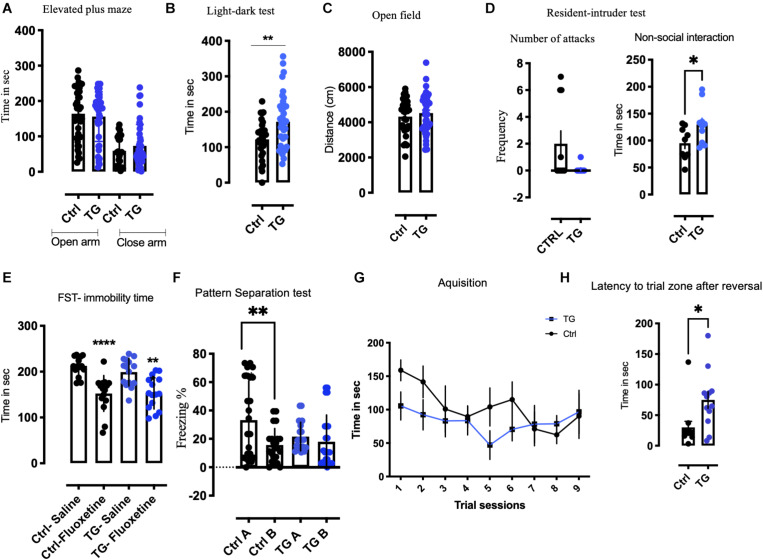
Transgenic animals displayed anxiolytic like, non-aggressive interaction, altered pattern separation, and reversal learning in a battery of behavioral tests. **(A)** In the Elevated plus maze (EPM), no significant change was observed in the time spent in open or closed arm in both the groups (*N* = 30/group). **(B)** In the light-dark test (LD), time spent in the light by the TG animals was significantly higher than controls (*N* = 30/group). **(C)** In the open field (OF), the total distance traveled remained unchanged between the groups (*N* = 30/group). **(D)** In resident intruder test (RI), number of attacks was unaffected between the genotypes, whereas significant non-social behavior was observed. The TG animals showed non-social defensive behavior compared with the controls (*N* = 12/group). **(E)** The Immobility time was reduced after acute fluoxetine in both genotypes subjected to forced swim test (FST). **(F)** In Pattern separation experiment paradigm (PS), the control animals exhibit pattern completion distinguishing context A from context B after 10 days of continuous exposure to both the context. The TG animals contextual pattern separation is inhibited (*N* = 20/group). **(G)** In Barnes maze test (BM), both the animals learned during acquisition. **(H)** The latency to target zone during reversal training was significantly increased in the TG animals. Student’s *t*-test, Two-way ANOVA, *p-value < 0.01, **p-value < 0.001, and ****p-value < 0.0001. Error bars represent Means ± SEM.

In the EPM, no significant difference in the time spent in the open or close arm was observed between the groups (Figure 3A and Supplementary Figure 3A). In the LD test, the time spent in light compartment was increased in the TG mice (Figure 3B). In the open field test, in spite of the increased activity seen in the metabolic cages, we observed equal amount of ambulation for both the groups in total distance moved and time spent in the center (Figure 3C and Supplementary Figure 3B). To measure if these animals showed signs of aggression, they were subjected to RI paradigm. The experiment animals were residents in their home cage. No significant change was observed between the groups in the number of attacks on the intruder mice (Figure 3D). Interestingly, the non-social exploration behavior characterized by digging, rearing and scanning the intruder was increased in the TG animals (Figure 3D). The social exploration which included contacts with the intruder such as sniffing, chasing and climbing was unchanged (Supplementary Figure 3C). The acute effect of antidepressant fluoxetine was then assessed by the forced swim test. The drug fluoxetine was administered intraperitoneally 30 min before the test. Both groups responded similarly with decreased immobility (Figure 3E) suggesting that both the controls and TG animals responded to acute fluoxetine (*p* < 0.0001).

In a separate cohort of animals, we subjected the animals to a PS paradigm of fear conditioning (Figure 3F). The baseline activity of all the animals was analyzed before proceeding with the behavior protocol. We used both males and females in this experiment and found similar effects in both sexes. After 10 days of the training protocol, the control animals exhibited a significant pattern separation, while the TG animals showed no pattern separation by two-way ANOVA [*F*(24,64) = 2.167, *p* = 0.007] (Figure 3F). A detailed analysis of everyday freezing of both groups is shown in Supplementary Figure 4. This phenomenon of incomplete pattern separation is attributable to impairment in memory consolidation.

In order to characterize the effect of the TrkB deletion on spatial learning and memory, we performed a BM test. During training, the animals learned to find the escape box equally, irrespective of the genotype (Figure 3G). When after the initial training the goal was moved to the diagonally opposite quadrant, the TG animals needed significantly longer time to reach the new goal (Figure 3H), indicating impaired cognitive flexibility in the TG mice.

### Increased Newborn Cells and Altered Mature Neuron Markers in TG Mice

**FIGURE 4:**
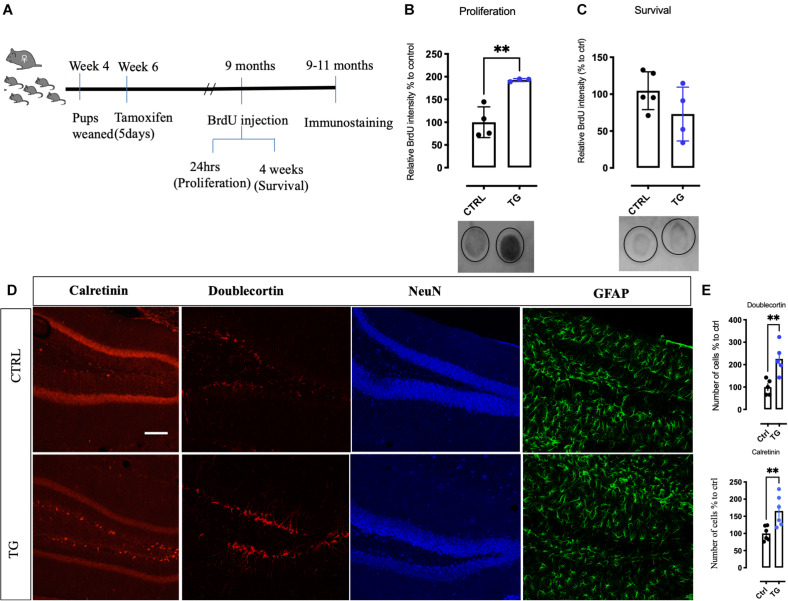
Increased new born precursor cells and altered neuronal markers in the hippocampus of the transgenic mice. **(A)** A schematic representation of time points for BrdU injection to study proliferation and survival of hippocampal neurons. **(B)** Quantitation of new-born neurons with BrdU incorporation in the transgenic animals by dot-blot. In samples collected for proliferation, TG animals showed a robust increase. **(C)** In the survival samples, no difference in BrdU incorporation was observed in both the groups. **(D)** Markers for different stages of neurogenesis was studied in both the groups. In the DG of the HC, DCX, and Calretinin was increased in the transgenic animals, however, NeuN and GFAP immunoreactivity was found to be unaltered. **(E)** Quantitative analysis of the neurons for DCX and Calretinin shows significant increase in the TG animals. Scale bar represents 100 μm, *N* = 4/group, Unpaired *t*-test **p-value < 0.01. Error bars represent Means ± SEM. HC-Hippocampus, DCX-Doublecortin, DG-Dentate gyrus.

The 5-HT innervation is known to regulate neurogenesis in adult hippocampus (Gould, 1999). We therefore investigated proliferation and survival of cells in the DG of TG mice using the method of BrdU incorporation into the DNA (Wu and Castrén, 2009; Casarotto et al., 2021). A schematic representation of the experimental timeline for BrdU administration and immunostaining is represented in Figure 4A. One day after BrdU administration, we observed increased precursor cell proliferation in the TG mice when compared to controls (Figure 4B). However, 4 weeks after BrdU injection, the levels of BrdU were greatly reduced and no significant difference between the genotypes was observed (Figure 4C), indicating that the excessively produced newborn cells failed to survive (Malberg et al., 2000; Santarelli et al., 2003). Consistently, the number of doublecortin (DCX, a marker of early post-mitotic neurons) and calretinin (marker of late post-mitotic neurons) positive neurons were significantly increased in the DG of the TG mice (Figures 4D,E). Thus, absence of TrkB receptors in the 5-HT neurons projecting to the HC increased the rate of cell proliferation but did not influence the long-term survival of hippocampal progenitors.

## Discussion

In this study, we have investigated the role of TrkB expression in Tph2 expressing 5-HT neurons. Our results suggest that loss of the TrkB receptor in the 5-HT neurons increases 5-HT levels, thereby regulating neuronal plasticity and behavior. Reduction of TrkB in 5-HT neurons increase proliferation, but not long-term survival, of hippocampal cells that is consistent with increase in immature neuronal markers such as doublecortin and calretinin in the transgenic animals.

Previous studies have revealed a significant role for BDNF signaling in the early differentiation of 5-HT neurons (Galter and Unsicker, 2000a, b). Furthermore, excess 5-HT during development impairs cortical differentiation (Gaspar et al., 2003; Deneris and Gaspar, 2018). Deletion of monoamine oxidase A (MAOA) in transgenic mice increases 5-HT levels and interferes with the formation of visual and somatosensory maps (Cases et al., 1996; Salichon et al., 2001), and this phenotype was further accentuated when TrkB levels were reduced (Vitalis et al., 2013). To avoid potentially deleterious effects of TrkB loss on developing 5-HT neurons, we have used a conditional deletion of TrkB from these neurons in the adulthood and the early development of the 5-HT neurons is therefore intact. We did not observe any obvious loss of 5-HT neurons and the expression of *Tph2* was in fact increased, suggesting that BDNF does not appear to be a critical survival factor for adult serotonin neurons. Our data indicate that BDNF through TrkB plays an important role in the regulation of 5-HT neurons and is likely a key element in the control of its function.

Our data suggest that BDNF signaling through TrkB plays a major role in the proper functioning of the 5-HT neurons. Tph2, the 5-HT synthesizing enzyme in the brain (Walther et al., 2003) was increased in the TG mice which is consistent with increased 5-HT levels both in the MB as well as in the projection areas of these neurons in the PFC. Although the expression of most of the 5-HT receptors remained unchanged, the *5ht2b* transcripts were significantly lower in the PFC of TG mice. 5HT2b agonists show antidepressant like effect and works in modulating serotonergic tone like selective serotonin reuptake inhibitors (SSRIs) (Diaz et al., 2016). In mice, 5HT2b receptors have been reported to regulate extracellular 5-HT levels (Callebert et al., 2006). However, the exact role of 5HT2b receptors in the increased serotonin levels observed in the TG mice requires further investigation.

Excess 5-HT, especially during development, produces structural and functional abnormalities that are long-lasting (reviewed in Gaspar et al., 2003; Deneris and Gaspar, 2018). Even if 5-HT levels were increased, the main 5-HT metabolite 5-HIAA was not significantly enhanced and the ratio of 5-HIAA/5-HT, a measure of 5-HT turnover (Valzelli and Bernasconi, 1979) was decreased. Several other factors, in addition to increased synthesis, including lowered degradation rate or increased storage capacity, may have contributed to increased serotonin levels. Our data suggest increased serotonergic tone in the TG mice, although additional studies with microdialysis and *in vivo* voltammetry will be needed to investigate the role of TrkB deletion in 5-HT release in more detail.

The serotonergic system is known to regulate appetite, and drugs that promote 5-HT release have been used for appetite reduction (Halford et al., 2005). We found that mice with loss of TrkB signaling in Tph2 expressing 5-HT neurons show significantly reduced body weight. Tph2 knockout mice lacking brain 5-HT have reduced weight at birth but their body weight normalizes during adulthood (Mosienko et al., 2015). Furthermore, we found that BDNF was increased in the TG mice. BDNF has been suggested to be one of the central factors regulating satiety in brain (Rios, 2013). Intraventricular BDNF administration reduces feeding and brain-wide reduction in the levels of BDNF or TrkB increases appetite and food intake, presumably acting at hypothalamic level, increasing body weight (Rios et al., 2001; Yeo et al., 2004; Gray et al., 2006; Rios, 2013). However, this reduced body weight is not produced by decreased appetite, as food consumption in the TG mice was actually increased, which suggest that the loss of weight was not a consequence of increased BDNF expression. Apparently, observed hyperactivity and increase in metabolic rate in mice with loss of TrkB in 5-HT neurons are sufficient to compensate the increased food intake. These data indicate that the effects of TrkB signaling and 5-HT on food intake and metabolic activity are complex and dependent on the brain regions being affected.

Adachi et al. (2017) recently reported that a virally induced loss of TrkB in the dorsal raphe region also increased aggression. In spite of high 5-HT levels and hyperactivity mice deficient of TrkB specifically in 5-HT neurons showed normal phenotype in tests assessing anxiety and aggression. Furthermore, we observed a normal response to fluoxetine in the forced swim test in the TG mice, which is in contrast to the finding of Adachi et al. (2017) who reported a loss of responsiveness to fluoxetine in their mice. Our recent data show that mice where TrkB was deleted using an En1-cre promoter that is active in the midbrain region covering, but not confined to, the raphe nuclei, show increased aggression (Sahu and Castrén, unpublished). Since Adachi et al. (2017) used a local midbrain injection of cre-expressing viruses to delete TrkB, it is possible that deletion of TrkB in cells next to 5-HT neurons, such as GABAergic interneurons, might mediate the aggressive phenotype observed by Adachi et al. and the antidepressant mechanism.

The 5-HT innervation is known to regulate the proliferation of hippocampal precursor cells (Deneris and Gaspar, 2018). Chronic antidepressant treatment increases neurogenesis (Malberg et al., 2000; Santarelli et al., 2003) and long-term survival of these newborn neurons is regulated by BDNF signaling (Sairanen et al., 2005). We found a significant increase in hippocampal cell proliferation and early differentiation of newborn DG neurons, as indicated by increased BrdU incorporation and doublecortin as well as calretinin positive neurons, although the latter finding awaits confirmation with stereological methods. However, their long-term survival was at a wildtype level, which is consistent with the notion that long-term survival requires activity-dependent incorporation into hippocampal networks (Castren and Hen, 2013). The failure of long-term survival is also consistent with the impairment in cognitive flexibility and alterations in pattern separation, processes that are thought to be dependent on the hippocampal function,

We have previously observed that complete loss of TrkB in zebrafish has a major impact on the development of 5-HT and DA neurons (Sahu et al., 2019). Current findings indicate that the effects of TrkB signaling in the mammalian 5-HT neurons are predominantly at a functional level. Our data demonstrate that deleting a receptor in a circumscribed group of neurons can have widespread cell non-autonomous *trans* effects in many parts of the adult central nervous system. Through increased synthesis of 5-HT, lack of TrkB in these neurons significantly impacts on the maturation of hippocampal neurons and consequently the animal behavior. These findings underline the previously implicated close connectivity between neurotrophins and the 5-HT system (Mattson et al., 2004; Martinowich and Lu, 2008).

## Data Availability Statement

The original contributions presented in the study are included in the article/Supplementary Material, further inquiries can be directed to the corresponding authors.

## Ethics Statement

The animal study was reviewed and approved by the Animal Ethical Committee of Southern Finland (Finland: ESAVI/10300/04.10.07/2016 and ESAVI/38503/2019).

## Author Contributions

MS and EC planned the experiments and wrote the manuscript. MS, YP-B, MP, AS, OB, and KK performed the behavioral and biochemical experiments. TP performed the HPLC analysis on the animals. All authors contributed to the article and approved the submitted version.

## Conflict of Interest

The authors declare that the research was conducted in the absence of any commercial or financial relationships that could be construed as a potential conflict of interest.

## Supplementary Material

Supplementary Figure 1 Confirmation of cre activation and Tph2 expression by tamoxifen injection in comparison with the corn oil group. The arrow heads represent the expression location in the cre positive animals. In the corn oil injected animals only Tph2 immunoreactivity is observed. The scale bar = 5 mm.

Supplementary Figure 2 **(A)** Western blotting with TrkB antibody in the hippocampus **(a)** and hypothalamus **(b)** of the Ctrl and TG mice, GAPDH antibody staining of the same gel was used for normalization (*N* = 3/group). Histograms show quantitated TrkB bands normalized by GAPDH for hippocampus **(c)** and hypothalamus **(d)**. **(B)** HPLC of other brain regions for Dopamine, serotonin, and 5HIAA/5-HT ratio remains unchanged between the groups.

Supplementary Figure 3 **(A)** Parameters for number of open arm entries and time spent in open arm remains unchanged between the groups. **(B)** No change with the time spent in the center of the open field. **(C)** Social interaction time in the resident intruder paradigm is unaffected between the groups.

Supplementary Figure 4 Freezing percentage of every day tracking in the PS test for both the genotypes. **(A)** Controls and **(B)** TG animals.
