# Supplementary figures and images for: Depletion of TrkB Receptors From Adult Serotonergic Neurons Increases Brain Serotonin Levels, Enhances Energy Metabolism and Impairs Learning and Memory
Source: Front Mol Neurosci. 2021 Apr 15;14:616178. doi: 10.3389/fnmol.2021.616178 (PMC8082189; doi:10.3389/fnmol.2021.616178)

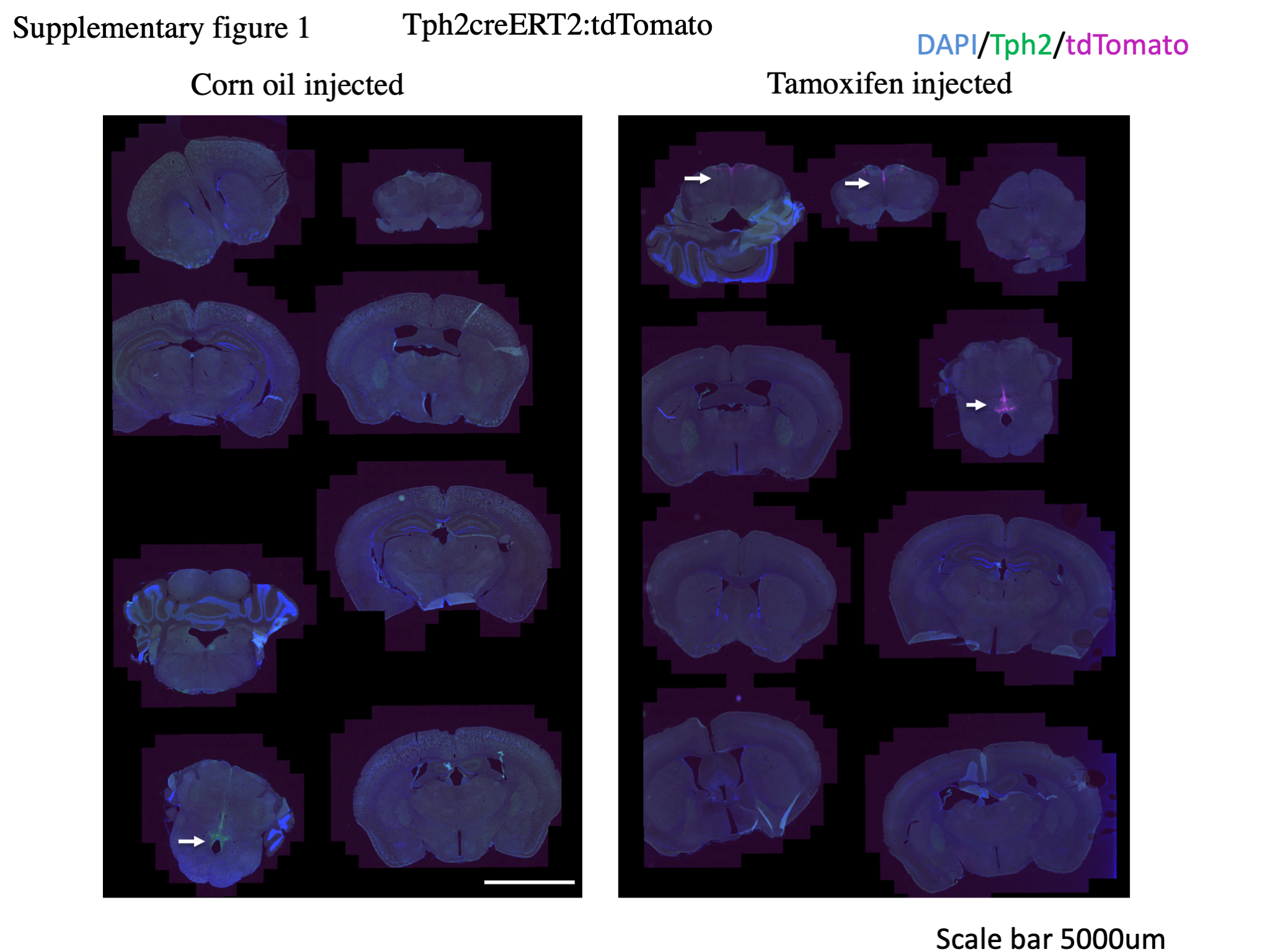

Supplement: Supplementary Figure 1 — Confirmation of cre activation and Tph2 expression by tamoxifen injection in comparison with the corn oil group. The arrow heads represent the expression location in the cre positive animals. In the corn oil injected animals only Tph2 immunoreactivity is observed. The scale bar = 5 mm. [file Image_1.TIFF]

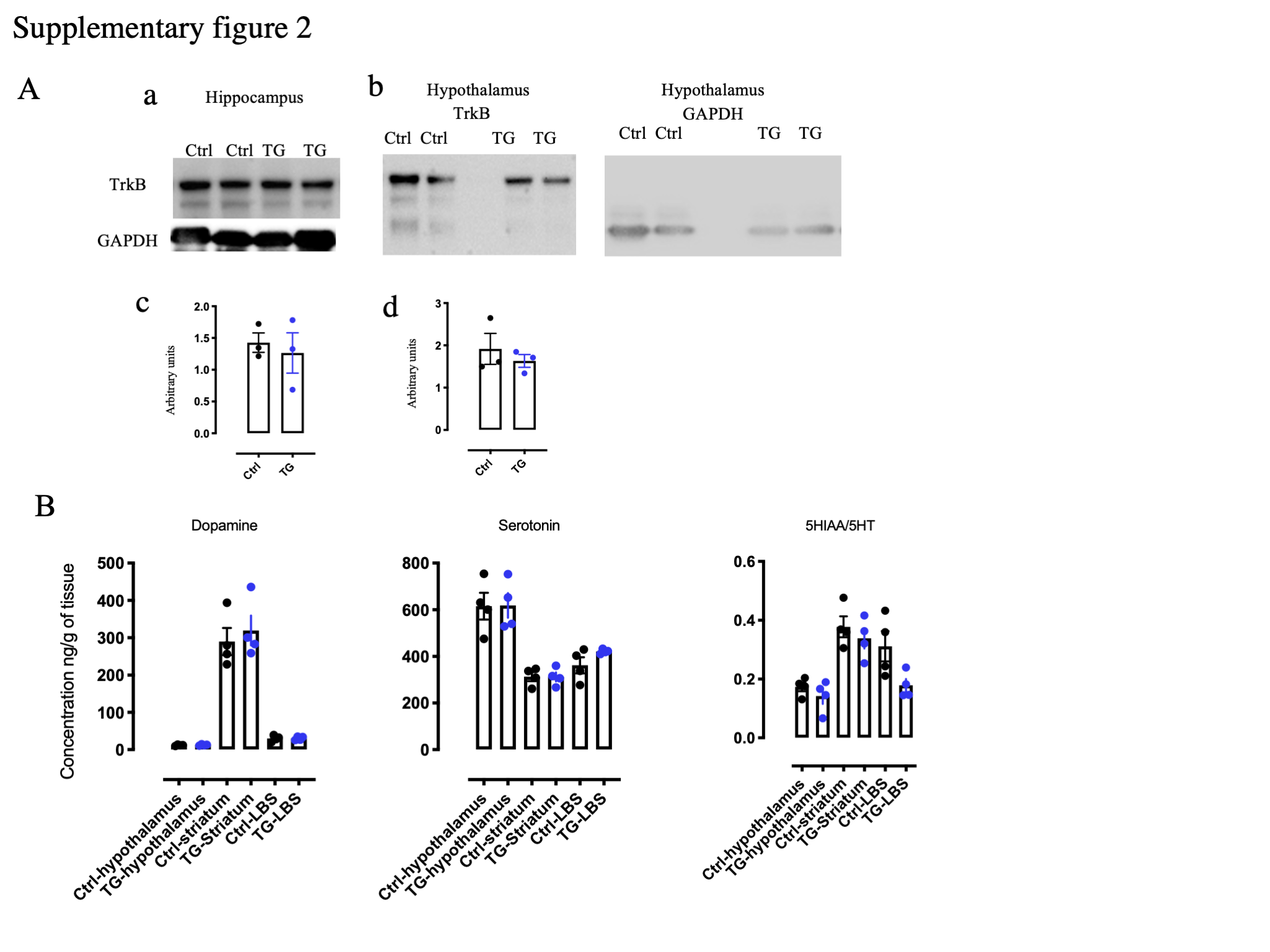

Supplement: Supplementary Figure 2 — (A) Western blotting with TrkB antibody in the hippocampus (a) and hypothalamus (b) of the Ctrl and TG mice, GAPDH antibody staining of the same gel was used for normalization (N = 3/group). Histograms show quantitated TrkB bands normalized by GAPDH for hippocampus (c) and hypothalamus (d). (B) HPLC of other brain regions for Dopamine, serotonin, and 5HIAA/5-HT ratio remains unchanged between the groups. [file Image_2.tiff]

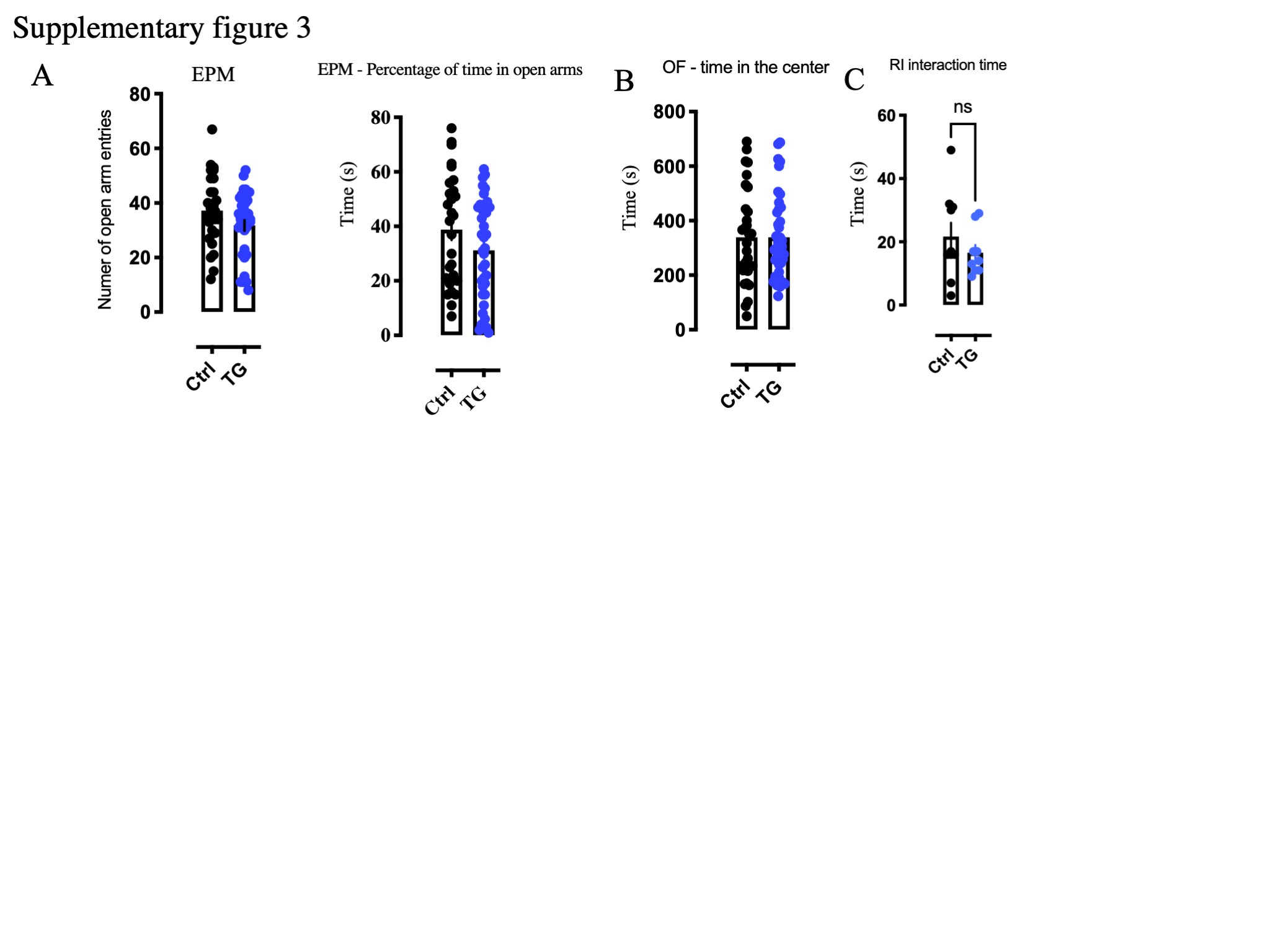

Supplement: Supplementary Figure 3 — (A) Parameters for number of open arm entries and time spent in open arm remains unchanged between the groups. (B) No change with the time spent in the center of the open field. (C) Social interaction time in the resident intruder paradigm is unaffected between the groups. [file Image_3.tiff]

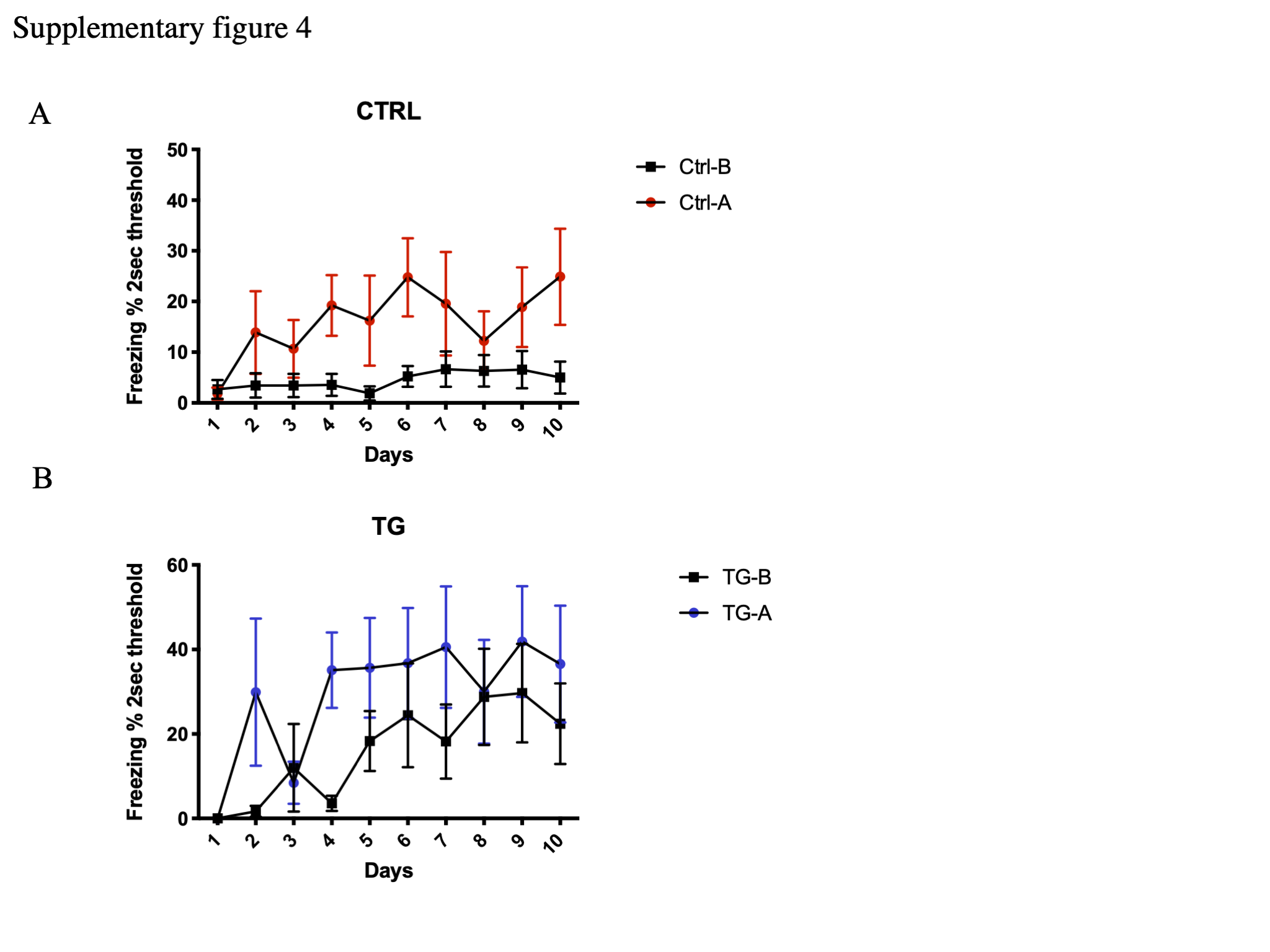

Supplement: Supplementary Figure 4 — Freezing percentage of every day tracking in the PS test for both the genotypes. (A) Controls and (B) TG animals. [file Image_4.tiff]
